# Comparison of individual and neighbourhood socioeconomic status in case mix adjustment of hospital performance in primary total hip replacement in Sweden: a register-based study

**DOI:** 10.1186/s12913-020-05510-0

**Published:** 2020-07-10

**Authors:** Johan Mesterton, Carl Willers, Tobias Dahlström, Ola Rolfson

**Affiliations:** 1 Department of Learning, Informatics, Management and Ethics, Medical Management Centre, Karolinska Institutet, Tomtebodavägen 18 A, 171 77 Stockholm, Sweden; 2 Ivbar Institute AB, Stockholm, Sweden; 3 Department of Neurobiology, Care Sciences and Society, Karolinska Institutet, Stockholm, Sweden; 4 Department of Public Health and Caring Sciences, Health Services Research, Uppsala university, Uppsala, Sweden; 5 Department of Orthopaedics, Institute of Clinical Sciences, Sahlgrenska Academy, University of Gothenburg, Gothenburg, Sweden; 6 The Swedish Hip Arthroplasty Register, Centre of Registers Västra Götaland, Gothenburg, Sweden

**Keywords:** Performance measurement, Case mix adjustment, Risk adjustment, Total hip replacement, Socioeconomic status

## Abstract

**Background:**

Case mix adjustment is a pre-requisite for valid measurement of healthcare performance and socioeconomic status (SES) is important to account for. Lack of information on individual-level SES has led to investigations into using a proxy for SES based on patient area of residence. The objective of this study was to use neighbourhood SES for case mix adjustment of performance indicators in total hip replacement (THR) in Sweden, and to compare with use of individual SES.

**Methods:**

Data from patient administrative systems and the Swedish Hip Arthroplasty Register were extracted for all patients undergoing THR in four Swedish regions. For each subject, individual data and neighbourhood data on country of birth, educational level, and income were provided by Statistics Sweden. Three variables were selected for analysis of performance; EQ-5D, hip pain and length of stay (LoS). In addition to socioeconomic information, several important clinical characteristics were used as case mix factors. Regression analysis was used to study each variable’s impact on the three outcome variables and model fit was evaluated using mean squared error.

**Results:**

A total of 27,121 patients operated between 2010 and 2016 were included in the study. Both educational level and income were higher when based on neighbourhood information than individual information, while proportion born in Sweden was similar. Higher SES was generally found to be associated with better outcomes and lower LoS, albeit with certain differences between the different measures of SES. The predictive ability of the models was increased when adding information on SES to the clinical characteristics. The increase in predictive ability was higher for individual SES compared to neighbourhood SES. When analysing performance for the two providers with most diverging case mix in terms of SES, the inclusion of SES altered the relative performance using individual as well as neighbourhood SES.

**Conclusions:**

Incorporating SES improves case mix adjustment marginally compared to using only clinical information. In this patient group, geographically derived SES was found to improve case mix adjustment compared to only clinical information but not to the same extent as actual individual-level SES.

## Background

Routine performance measurement serves to evaluate the extent to which health services meet performance targets that include achievement of optimal health outcomes, responsiveness to patient preferences, equity of access, and efficiency in the delivery of services [1, 2]. Transparency around hospital performance is gaining interest as a measure to improve healthcare and is now being addressed by OECD [3]. Benchmarking across nations, regions and hospitals is steadily increasing and may be used as a lever to identifying best practices and enabling systematic evaluation of performance. In 2013, several Swedish regions together with the Ministry of Health launched the Sveus research project, with the objective to create benchmarking between regions and hospitals for several different patient groups and thereby enabling the spreading of best practices and continuous improvement.

Case mix adjustment is a prerequisite for interhospital comparisons because it adjusts results for differences in underlying patient populations, thus enabling “apples-to-apples” comparisons [4]. Including socioeconomic status (SES) information in such adjustment is important for ensuring fair comparisons of performance given that it has a strong impact on health [5]. In the United States, a 2014 report from the National Quality Forum recommended that sociodemographic factors should be adjusted for in performance measures [6]. Ultimately, the importance of SES on outcomes and costs implies that adjustment for SES in comparisons between providers may promote health equity. Health equity across socioeconomic groups is also an explicit goal in policy documents from the United Nations [7], the European Union [8] and stated in Swedish law [9]. Despite this, information on SES has rarely been incorporated in case mix adjustment, and this is likely largely due to difficulty in obtaining these data. Individual-level data on individual SES is available in national registers in Sweden with universal coverage, but these can only be used for research under granted ethical permission [10]. Hence, the information cannot be integrated into administrative systems or electronic health records and can consequently not be used for continuous analysis of performance by payers or providers. The challenge in obtaining this type of information is seen also in many other countries and the absence of individual-level socioeconomic data has led to investigations into using a proxy based on information about the geographic area the individual resides in [11, 12]. The impact of neighbourhood socioeconomic status (NSES) on both health outcomes and costs of care has been investigated in numerous studies, and it has been used both as a substitute and as a complement to individual SES (ISES) [13–16].

Total hip replacement (THR) is a common elective operation. The incidence of total hip replacement in Sweden is among the highest in the world [17]. The incidence is projected to keep rising, as demographics change with an older population [18]. Approximately 0.35% of individuals over 40 years of age receive a total hip replacement every year in Sweden, and approximately 3.4% of the same group have at least one hip prosthesis [17], which highlights the importance of systematic measurement of performance related to this procedure. There is a long tradition in Sweden with collecting and measuring results after THR; the Swedish Hip Arthroplasty Register (SHPR) was founded in the 1970’s and has a coverage rate of 98% for THR [17]. SHPR was also one of the early adopters of collecting patient-reported outcome measures.

The aim of this study was to evaluate the use of NSES for case mix adjustment of performance indicators in THR in Sweden, and to compare usefulness of such an aggregated risk adjustment measure in comparison to the use of ISES.

## Methods

### Study population and data sources

All THR performed during 2010–2016 in four Swedish regions (Dalarna, Skåne, Västra Götaland, Uppsala) were considered eligible for inclusion in the study population, based on registration in both the national quality register (SHPR) and in the regional patient administrative systems (PAS). The regions included were supposed to cover relatively urban as well as more rural areas of Sweden. Cases were selected based on procedure codes for primary total hip replacement (NFB29, NFB39, NFB49, NFB62, NFB99). Data with personal identification number was sent to Statistics Sweden who replaced the identification number with an anonymized number used for linkage. Ethical permission for the study was granted by the regional ethical review board in Stockholm (reference number 2017/746–31/5).

PAS data contain administrative information regarding the patients and all their care contacts in the region, including diagnosis (ICD-10) and procedure codes (KVÅ and KKÅ, the Swedish version of the Nomesco classification of surgical procedures, NCSP). SHPR contains more detailed information on the actual procedure as well as several patient-reported outcome measures, including health status in terms of EQ-5D, as well as hip pain before and after the surgery. Statistics Sweden collects individual-level socioeconomic data for the entire Swedish population, including information on highest educational level, country of birth and disposable income. This information was used to get information about actual (individual) SES for the study population. Based on this information, Statistics Sweden may also provide information on average socioeconomic information by geographical area in Sweden. Each geographic area can be as small as 250*250 m in densely populated areas and 1000*1000 m in sparsely populated areas. To ensure confidentiality, the NSES data for a specific area is scrambled by Statistics Sweden prior to delivery of data if less than four individuals live in that area. Information about each patient’s location of residence (according to the geographical information system, GIS, coordinates) was also available from Statistics Sweden. The average SES per geographical area was matched to each individual via their geographic area of residence, resulting in information on both ISES as well as NSES for each individual in the study population.

### Study variables

The three performance indicators selected were health-related quality of life (EQ-5D) score and hip pain score at 12 months post-operatively, and LoS [2]. EQ-5D and hip pain are both relevant for understanding performance in terms of quality of the surgery and care provided. These measures refer to levels reported at 12 months after surgery. LoS in conjunction to the hip replacement inpatient care episode was included as it is a significant cost driver related to surgery. Data on hip pain was treated in line with SHPR; as the Likert scale was introduced for reporting by the register during 2016, patient reported pain on a visual analogue scale (0–100) has been transposed to a Likert scale (1–5) to enable comparison over time [19].

Several explanatory variables were included in the analysis. In addition to clinical characteristics – age, sex, comorbidity, Health-Related Quality of Life (EQ-5D) at baseline, hip pain at baseline, bilateral surgery, and previous contralateral hip prosthesis – a number of socioeconomic indicators were used. Baseline levels of EQ-5D and hip pain were included as post-surgery levels were expected to be correlated to pre-surgery levels. Based on availability of information on SES, the following indicators were used: highest educational level – 9 years or less in school (low), 10–12 years in school (medium), and more than 12 years in school (high) – born in Sweden, and income (total earned income for ISES and area median income for NSES). These are all presented in Table 1.

**Table 1:** Baseline characteristics, including clinical characteristics, individual and neighbourhood SES, and outcomes

Variable	Mean/Proportion	Std deviation
**Clinical characteristics**		
Age	69.3	10.1
Sex, proportion men	43%	
Comorbidity - Elixhauser index score	1.3	1.3
EQ-5D, at surgery	0.26	0.32
Pain, at surgery	2.5	0.8
Bilateral surgery	1%	
Previous hip prosthesis	9%	
**Individual socioeconomic characteristics**		
Income per year, Swedish krona	242,593	157,396
Born in Sweden	86%	
Highest educational level: high	25%	
Highest educational level: medium	41%	
Highest educational level: low	33%	
**Neighbourhood socioeconomic characteristics**		
Income per year, Swedish krona	258,834	65,990
Born in Sweden	86%	
Highest educational level: high	35%	
Highest educational level: medium	44%	
Highest educational level: low	20%	
**Outcome variables**		
Length of stay, orthopaedic unit, at surgery	4.0	2.8
EQ-5D, one-year follow-up	0.63	0.40
Pain, one-year follow-up	0.6	0.9

### Statistical analysis

The correlation between neighbourhood income and individual income was estimated using Pearson’s correlation coefficient. Regression analysis was used to analyse the impact of each predictor on the outcome of interest, with different models applied depending on the nature of the dependent variable. For both EQ-5D (measured on a scale from − 0.594 to 1 [20]) and hip pain (measured on a scale from 1 to 5), ordinary least squares regression was used. Length of stay in days, based on the dates of admission and discharge, was modelled using negative binomial regression. The resulting prediction models were used to calculate a predicted value for each patient and indicator. To evaluate model fit the Mean squared error (MSE), i.e. mean squared difference between predicted and actual value, was used. As a sensitivity analysis, the Akaike Information Criterion was also used. However, because the results were very similar between the two, we report only MSE which holds a more intuitive interpretation and can be directly related to the scale of the dependent variable.

To highlight the impact of adjusting for socioeconomic information when benchmarking outcomes, the two caregiving units that deviated the most (up and down, respectively) from the average in terms of SES of their patients were selected for analysis. For each patient and indicator, a prediction was made and the expected level for each hospital, for each indicator, was calculated by applying the average of all patients’ predicted values. This procedure was carried out for the different prediction models including different sets of predictors, resulting in different expected values depending on the amount of information included in the prediction model. Hospital performance for the different indicators was calculated by taking the ratio of the observed and the expected value. Indicators for which a lower value is desired (length of stay and pain level) the performance values were inverted so that a higher value would consistently be interpreted as better performance.

## Results

### Descriptive statistics

**Fig. 1:**
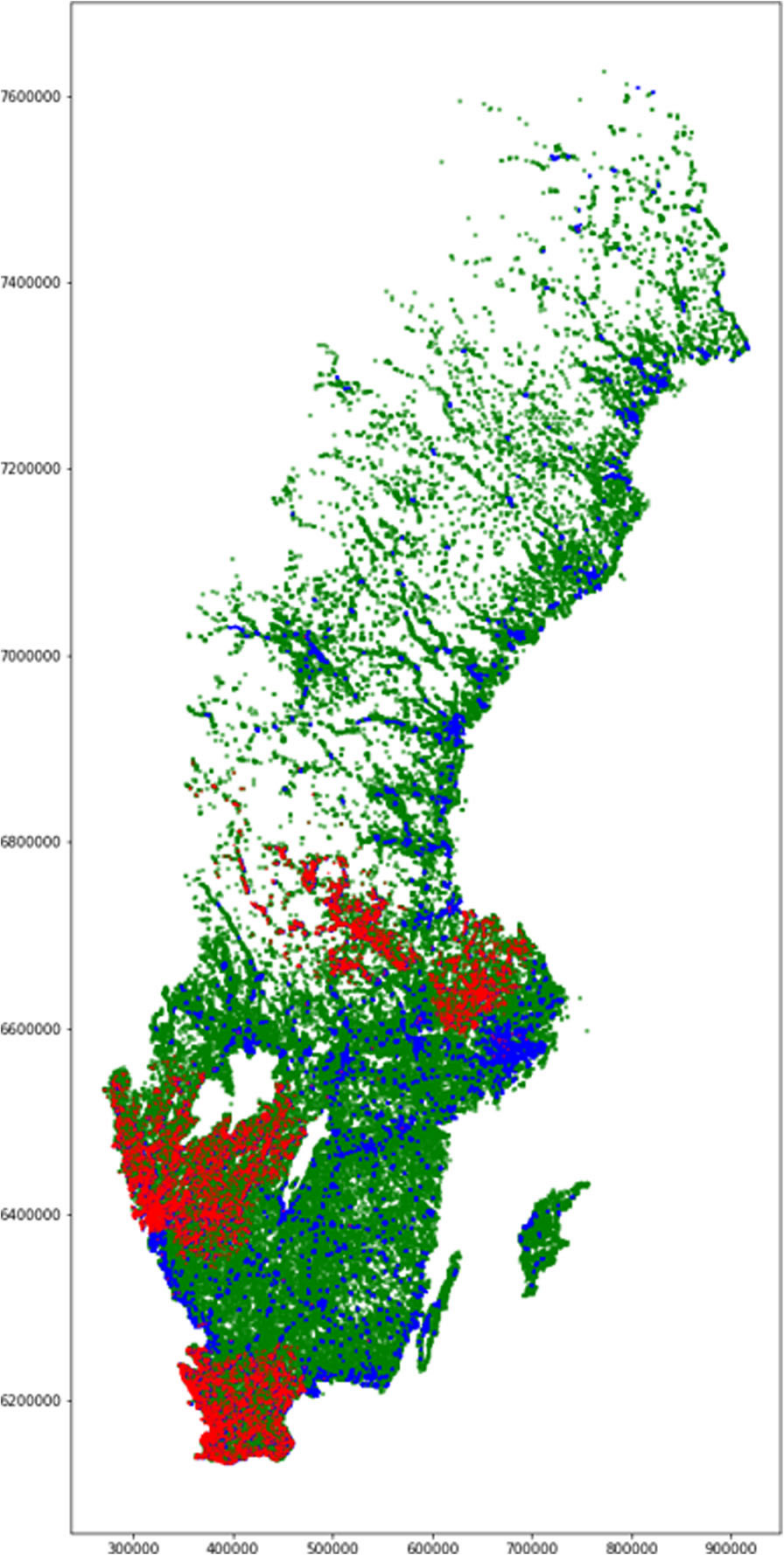
Plot of each coordinate for which neighbourhood socioeconomic data was available

A total of 27,121 patients operated between 2010 and 2016 were included in the study. Additional file 1 presents number of patients per year and region. Figure 1 presents a visual representation of each geographical area for which neighbourhood SES was available from Statistics Sweden. The green squares represent sparsely populated areas, the blue squares represent densely populated areas and the red dots are geographical areas in which patients in the study population resided, clustered into the four regions included in the study.

Table 1 describes the study population in terms of clinical and socioeconomic characteristics. 43% were men, and almost every tenth subject had a history of previous total hip replacement in the contralateral joint. Neighbourhood educational level was higher than individual educational level. Knowing that educational levels are higher in lower age groups, this difference is likely due to demographic differences between patients undergoing THR compared to the general population in their neighbourhoods. The proportion with > 12 years of education was higher in the neighbourhoods (35%) than in the study population (25%). The Pearson correlation between neighbourhood income and individual income amounted to 0.28.

### Effect of clinical characteristics and SES on quality of life, pain and length of stay

Table 2 presents the relationship between the different baseline characteristics of patients and the three different performance indicators investigated. Higher patient age and comorbidities were both associated with lower post-operative EQ-5D and pain, and also with longer LoS. Men reported higher post-operative EQ-5D and lower pain. The effects of these clinical characteristics were similar when also adjusting for individual and neighbourhood SES. Higher individual income was associated with higher post-operative EQ-5D, lower post-operative pain and shorter LoS. These relationships were not observed for neighbourhood income. Born in Sweden was associated with higher EQ-5D, lower pain and shorter LoS. Interestingly, the effects were stronger for neighbourhood SES (proportion of patients in the neighbourhood living in Sweden) than for individual SES (based on where the patient was actually born). Educational level did not have any significant association with post-operative EQ-5D. However, high educational level (irrespective of whether derived from the individual or that of the neighbourhood) was associated with lower pain and shorter LoS.

**Table 2:** Impact of clinical and socioeconomic factors on health-related quality of life, hip pain and length-of-stay

	EQ 5D			Hip pain			LoS		
	Clinical characteristics	Clinical characteristics and individual SES	Clinical characteristics and neighbourhood SES	Clinical characteristics	Clinical characteristics and individual SES	Clinical characteristics and neighbourhood SES	Clinical characteristics	Clinical characteristics and individual SES	Clinical characteristics and neighbourhood SES
Predictor	estimate (*p*-value)	estimate (*p*-value)	estimate (*p*-value)	estimate (*p*-value)	estimate (*p*-value)	estimate (*p*-value)	estimate (*p*-value)	estimate (*p*-value)	estimate (*p*-value)
**Constant**	0.802 (< 0.01)	0.707 (< 0.01)	0.753 (< 0.01)	0.863 (< 0.01)	1.189 (< 0.01)	1.121 (< 0.01)	0.736 (< 0.01)	0.961 (< 0.01)	1.014 (< 0.01)
**Baseline EQ-5D**	0.161 (< 0.01)	0.155 (< 0.01)	0.159 (< 0.01)						
**Baseline hip pain**				0.096 (< 0.01)	0.088 (< 0.01)	0.093 (< 0.01)			
**Age**	−0.001 (< 0.01)	− 0.001 (< 0.01)	− 0.001 (< 0.01)	0.002 (< 0.01)	0.001 (0.18)	0.002 (< 0.01)	0.014 (< 0.01)	0.012 (< 0.01)	0.014 (< 0.01)
**Male sex**	0.039 (< 0.01)	0.031 (< 0.01)	0.038 (< 0.01)	− 0.069 (< 0.01)	− 0.056 (< 0.01)	− 0.068 (< 0.01)	− 0.081 (< 0.01)	− 0.076 (< 0.01)	− 0.077 (< 0.01)
**Prior surgery**	0.025 (< 0.01)	0.024 (< 0.01)	0.026 (< 0.01)	− 0.085 (< 0.01)	− 0.083 (< 0.01)	− 0.085 (< 0.01)	− 0.117 (< 0.01)	− 0.115 (< 0.01)	− 0.117 (< 0.01)
**Number of comorbidities**	− 0.024 (< 0.01)	− 0.022 (< 0.01)	− 0.023 (< 0.01)	0.04 (< 0.01)	0.035 (< 0.01)	0.039 (< 0.01)	0.064 (< 0.01)	0.059 (< 0.01)	0.063 (< 0.01)
**Bilateral surgery**	0.037 (0.37)	0.042 (0.31)	0.039 (0.35)	0.102 (0.23)	0.088 (0.30)	0.101 (0.23)	0.543 (< 0.01)	0.528 (< 0.01)	0.536 (< 0.01)
**Income**		0.01 (< 0.01)	0.006 (0.24)		−0.02 (< 0.01)	0.02 (0.11)		−0.009 (< 0.01)	0.019 (0.01)
**Born in Sweden**		0.039 (< 0.01)	0.053 (0.01)		−0.146 (< 0.01)	− 0.182 (< 0.01)		− 0.113 (< 0.01)	−0.309 (< 0.01)
**Educational level high**		0.003 (0.70)	−0.008 (0.72)		−0.063 (< 0.01)	− 0.223 (< 0.01)		− 0.057 (< 0.01)	− 0.051 (0.13)
**Educational level medium**		0.002 (0.78)	−0.026 (0.28)		− 0.01 (0.45)	− 0.133 (0.04)		− 0.036 (< 0.01)	− 0.105 (< 0.01)
**Number of observations**	10,450	10,450	10,450	17,896	17,896	17,896	25,220	25,220	25,220

### Predictive ability of models using different sets of predictors

**Fig. 2:**
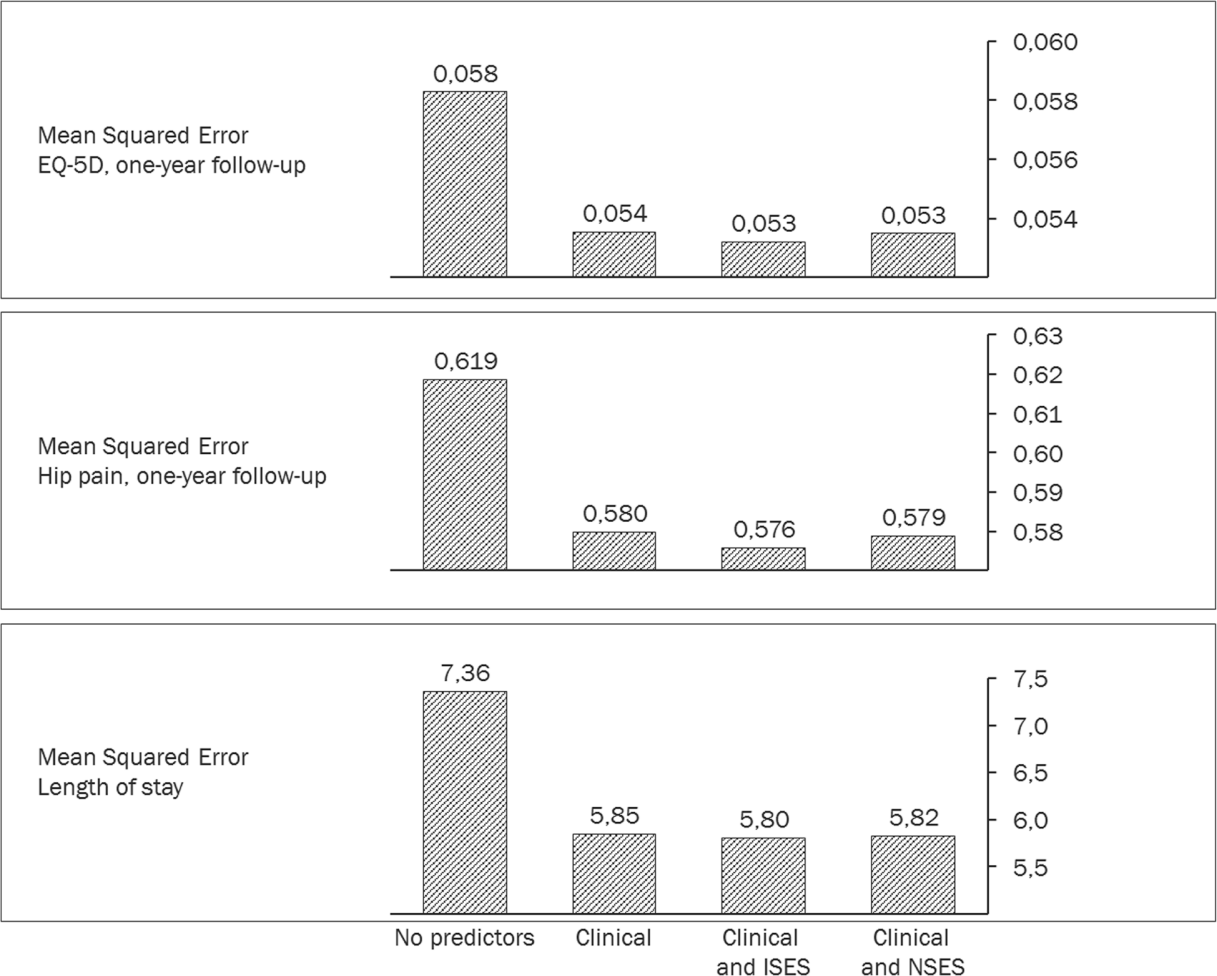
Predictive ability of models using different sets of predictors: Mean Squared Error of the different models. Lower value implies better predictive ability

Figure 2 shows the predictive ability of the different models. A model with no predictors was also included as reference point. Compared to using no predictors at all in the model, the ability of the models to predict post-operative EQ-5D, pain and LoS was significantly better when including the clinical characteristics of the patients. The improvement in predictive ability was least pronounced for EQ-5D and most pronounced for LoS. As the figure shows, the incremental improvement in predictive ability of adding SES to the clinical characteristics was relatively limited. Nevertheless, inclusion of individual SES was consistently associated with lower model error compared to only using clinical characteristics. Adding neighbourhood SES to the clinical characteristics also contributed to a slight improvement in predictive ability but the benefit was smaller than for individual SES.

### Impact on interhospital comparisons

**Fig. 3:**
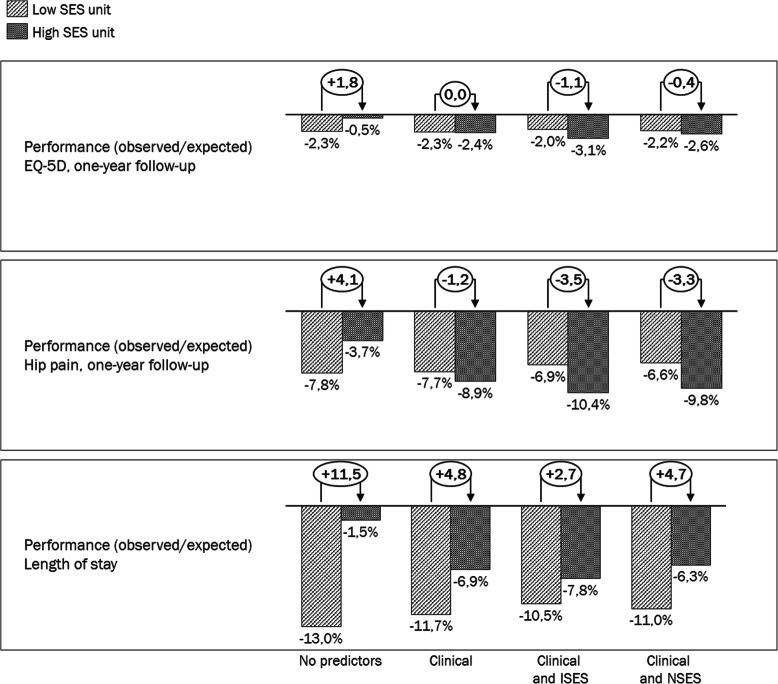
Impact of adjusting for SES when comparing the performance of units with different underlying patient populations. A negative value implies that the unit’s performance on that indicator is worse than expected (the inherent direction of the indicator is taken into account so that higher EQ-5D, lower pain and shorter LoS are interpreted as better performance). For each indicator there is one observed value per unit while there are four different expected values, one for each of the models used to derive an expected value. The numbers in the white circles describe difference in performance (in percentage points) between the units

For all three indicators investigated, the two selected units with the highest average SES and the lowest average SES showed worse results than the average (“unadjusted”) and also worse results when comparing the units’ observed value to the different expected values (based on clinical, clinical and individual SES versus clinical and neighbourhood SES, respectively) (Fig. 3). However, the comparison of performance between the 2 units did differ depending on whether case mix was accounted for or not. For all indicators, the unadjusted performance was worse for the “Low SES unit” compared to the “High SES unit”. When adjusting for differences in clinical characteristics the units had a similar performance in terms of EQ-5D, relatively similar performance in terms of pain (if anything the “High SES unit” performed worse than the “Low SES unit”) and the difference in performance regarding length of stay was smaller. When also accounting for patients’ SES there was a tendency that the “High SES unit” had worse performance for both EQ-5D and pain and the difference in length of stay was slightly less pronounced (the “High SES unit” performing better). Hence, for both EQ-5D and pain, the performance comparison between the “High SES unit” and the “Low SES unit” was altered through the inclusion of clinical characteristics and SES of the patients treated. For length of stay there was no reversal in performance following adjustment but the differences between the “High SES unit” and the “Low SES unit” diminished following adjustment for case mix.

Descriptive statistics of the patients treated at these units are included in the supplement (Additional file 2).

## Discussion

This study found that higher SES was associated with better outcomes and shorter length of stay, which both are important aspects of performance in THR. The impact of case mix adjustment on comparisons of performance differs between different indicators and the value of adding socioeconomic information to such adjustment also differs between indicators. The study illustrates that socioeconomic information adds an important perspective to interhospital comparisons, particularly regarding comparisons between units with highly diverging patient populations in terms of socioeconomic status. However, individual SES for entire patient populations is generally not available to use for such purposes. Nevertheless, with increasing amounts of data available, proxies of SES, such as neighbourhood SES, has the potential to add relevant information to benchmarking of performance. This study shows one such approach to socioeconomic adjustment of performance and presents the impact on interhospital comparisons in THR in Sweden.

The present study found a certain, albeit far from perfect, correlation between ISES and NSES. Compared to NSES, ISES was consistently found to have a greater, although marginal, impact on case mix adjustment. In line with the findings here, Marra et al. reported relatively marginal correlation between ISES and NSES [21]. Our findings also corroborate previous research which has shown a stronger impact of ISES, as compared to a proxy based on residential area, on health outcomes [22]. Furthermore, these results are in line with previous studies showing that aggregated measures of socioeconomic status do improve the understanding of health outcomes compared to no socioeconomic information at all [11, 12]. Using a similar methodological approach as the one used here, combining individual-level and neighbourhood-level official statistics, a Finnish study of patients with Diabetes type 2 found small-area based SES to be a relatively good substitute to individual-level information [23]. They found small-area based educational attainment to exhibit comparable predictive ability as individual educational attainment, while income did not have as consistent results between area-based and individual information.

Compared to using individual SES there are also potential advantages of using neighbourhood SES. For example, individual-level income declines dramatically at time of retirement from work, but this may not adequately reflect the change in socioeconomic status of the individual. However, if the retired individual does not change residency, his or her neighbourhood SES will remain the same following retirement. Thus, neighbourhood SES may be more stable indicator of SES in this regard. The correlation between individual and neighbourhood SES depends on how close the characteristics of the patient group are to those of the general population in the neighbourhood. A recent Swedish study of SES of patients with hip osteoarthritis found that SES was higher than in the general population after matching on sex, age and place of residence [24]. In the present study, the patients in the study population were significantly older than the general population in the neighbourhoods where the study population resided. This is not surprising given that THR is a procedure generally performed in an older (and often retired) population, which likely explains the lower income and educational level in the study population compare to that of the general population in their neighbourhoods. To achieve more common characteristics between patients and general population, there are possibilities to capture stratified NSES (e.g. SES by age group and/or sex). However, whilst increasing granularity, such stratification of individuals in small geographical areas may be challenging from an integrity perspective, and in the present study it would have resulted in a high degree of scrambling from the national authority delivering the NSES data. One remedy for this could be to apply larger geographical areas than in the current study to allow for increased stratification of the population. Further research is needed to determine whether best precision is achieved using small geographical areas with the entire population or larger geographical areas with stratification into subgroups.

Beside the characteristics of the patient population, several factors affect the outcome of THR. It is well known that factors such as the skill of the surgeon, prosthesis design, and a number of components related to the care process impact the outcomes after surgery. It is not to be expected that the case mix should explain the majority of the variation in outcomes; instead, case mix adjustment aims to remove potential impact of patient characteristics and thereby unveiling the effect of the provider [25]. Deviations in performance that remain after adjustment for case mix imply room for improvement and potential for spreading best practice within a clinical field. To gain insight on where best practice is actually performed, there is a need to eliminate differences between units caused by variations in case mix, including differences in socioeconomic dimensions.

Previous research has pointed to the importance of socioeconomic factors in joint replacement surgery regarding satisfaction and functional outcomes [26]. For outcomes in younger participants, the socioeconomic factors have been shown to have a stronger impact than the classical factors including demographic and implant factors [27]. In the therapeutic area studied here, neighbourhood SES and its association to outcomes is not as well studied as individual SES, but one study has found NSES to be associated with patient-reported outcomes after orthopaedic procedures [28]. Because THR is an elective procedure, it is important to consider the risk of selection bias when assessing the socioeconomic gradient. Here, we have investigated the impact of SES on outcomes and resource use in patients being operated. However, previous research suggest an effect also of SES on the probability of receiving the surgery at all [29, 30], which has not been analysed here.

Previous research suggests that the agreement between ISES and NSES differs between patient groups [21]. Given that THR is an elective surgery in an older patient population it is likely not appropriate to generalize the findings here to all other patient groups when it comes to the importance of SES, even though it is likely to be highly relevant regarding other orthopaedic elective procedures for degenerative conditions. In chronic patient groups, such as diabetes, with large individual responsibility, SES has been shown to be a very important factor [31], and it is possible that SES has a higher impact on interhospital comparisons there. The impact of socioeconomic adjustment, and the relevance of geographically derived measures of SES, in the analysis of performance in different types of patient groups is an area that warrants further research.

## Conclusions

Based on a relatively large sample of patients, for which detailed individual-level data on socioeconomic status was available, this study shows that incorporating socioeconomic information improves case mix adjustment slightly compared to using only clinical information. Geographically derived socioeconomic information was found to improve case mix adjustment compared to only clinical information but not to the same extent as actual individual-level socioeconomic status. Because THR is an elective surgery in an older patient population it is uncertain whether these findings are generalizable to patient groups with acute or chronic conditions or patient groups with other demographic profile. This would be an interesting area for further research.

## Supplementary information

**Additional file 1.** Overview of observations per year and region.

**Additional file 2.** Descriptive statistics of study population treated at the two caregiving units with the socioeconomic data deviating the most from average.

## Data Availability

Patient-level data may not be shared, because patient data regulations in Sweden and regulations of data holders do not allow for this.

## Abbreviations

EQ-5D: EuroQol Five-Dimension Scale
ICD-10: International Classification of Diseases, Tenth Revision
ISES: Individual-level Socioeconomic Status
LoS: Length-of-Stay
MSE: Mean Squared Error
NSES: Neighbourhood-level Socioeconomic Status
OECD: Organisation for Economic Co-operation and Development
PAS: Patient Administrative Systems
SES: Socioeconomic Status
SHPR: Swedish Hip Arthroplasty Register
THR: Total Hip Replacement
VGR: Region Västra Götaland
