# Supplementary material for: Comparison of individual and neighbourhood socioeconomic status in case mix adjustment of hospital performance in primary total hip replacement in Sweden: a register-based study
Source: BMC Health Serv Res. 2020 Jul 10;20:645. doi: 10.1186/s12913-020-05510-0 (PMC7353710; doi:10.1186/s12913-020-05510-0)
Supplement: Supplementary file 1 — Additional file 1. Overview of observations per year and region. [file 12913_2020_5510_MOESM1_ESM.docx]

Additional file 1. Overview of observations per year and region.

| **Year** | **Dalarna** | **Uppsala** | **Region Skåne** | **VGR** | **Total** |
| --- | --- | --- | --- | --- | --- |
| **2010** | 470 | 411 | 1399 | 1486 | 3766 |
| **2011** | 493 | 388 | 1379 | 1411 | 3671 |
| **2012** | 525 | 421 | 1321 | 1422 | 3689 |
| **2013** | 486 | 421 | 1414 | 1557 | 3878 |
| **2014** | 468 | 455 | 1432 | 1699 | 4054 |
| **2015** | 430 | 432 | 1449 | 1649 | 3960 |
| **2016** | 421 | 419 | 1539 | 1724 | 4103 |
| **All years** | 3 293 | 2 947 | 9 933 | 10 948 | 27 121 |
