# Supplementary material for: Comparison of individual and neighbourhood socioeconomic status in case mix adjustment of hospital performance in primary total hip replacement in Sweden: a register-based study
Source: BMC Health Serv Res. 2020 Jul 10;20:645. doi: 10.1186/s12913-020-05510-0 (PMC7353710; doi:10.1186/s12913-020-05510-0)
Supplement: Supplementary file 2 — Additional file 2. Descriptive statistics of study population treated at the two caregiving units with the socioeconomic data deviating the most from average. [file 12913_2020_5510_MOESM2_ESM.docx]

Additional file 2. Descriptive statistics of study population treated at the two caregiving units with the socioeconomic data deviating the most from average.

| **Variable** | **Mean/Proportion - Low SES unit** | **Mean/Proportion - High SES unit** |
| --- | --- | --- |
| **Clinical characteristics** |  |  |
| Age | 69.9 | 62.5 |
| Sex, proportion men | 41% | 47% |
| Comorbidity - Elixhauser index score | 1.1 | 0.9 |
| EQ-5D, at surgery | 0.42 | 0.39 |
| Pain, at surgery | 2.6 | 2.5 |
| Bilateral surgery | 0% | 2% |
| Previous hip prosthesis | 11% | 8% |
| **Individual socioeconomic characteristics** |  |  |
| Income per year, Swedish krona | 224,773 | 325,389 |
| Born in Sweden | 84% | 88% |
| Highest educational level: high | 19% | 38% |
| Highest educational level: medium | 35% | 38% |
| **Neighbourhood socioeconomic characteristics** |  |  |
| Income per year, Swedish krona | 251,481 | 282,329 |
| Born in Sweden | 85% | 87% |
| Highest educational level: high | 0.28 | 0.45 |
| Highest educational level: medium | 0.44 | 0.38 |
| **Outcome variables** |  |  |
| Length of stay, orthopaedic unit, at surgery | 4.5 | 4.0 |
| EQ-5D, one-year follow-up | 0.75 | 0.76 |
| Pain, one-year follow-up | 0.7 | 0.7 |
